# Taking a closer look at whole organisms

**DOI:** 10.7554/eLife.48340

**Published:** 2019-06-14

**Authors:** Noriko Ichino, Stephen C Ekker

**Affiliations:** Department of Biochemistry and Molecular BiologyMayo ClinicRochesterUnited States

**Keywords:** volumetric histology, whole-organism phenomics, 3D imaging, micro-CT, zebrafish, Zebrafish

## Abstract

By enabling researchers to image whole zebrafish with cellular resolution, X-ray histotomography will improve our understanding of the biological differences between individuals of the same species.

**Related research article** Ding Y, Vanselow DJ, Yakovlev MA, Katz SR, Lin AY, Clark DP, Vargas P, Xin X, Copper JE, Canfield VA, Ang KC, Wang Y, Xiao X, De Carlo F, van Rossum DB, La Riviere P, Cheng KC. 2019. Computational 3D histological phenotyping of whole zebrafish by X-ray histotomography. *eLife* **8**:e44898. doi: 10.7554/eLife.44898

For years, anyone trying to assess biological variation has had to choose between measuring whole organisms at low resolution or studying only parts of them at high (cellular) resolution. Tools such as micro-computed tomography (micro-CT) have made it possible to image whole organisms in three dimensions, but with limited imaging clarity and throughput. The field of histology has developed many approaches to study cells and tissues at high detail, but the methods used to prepare samples for viewing have restricted sample sizes; moreover, these techniques tend to work best in two not three dimensions. Now, in eLife, Keith Cheng of Penn State College of Medicine and colleagues – including Yifu Ding as first author – report how they have developed a technique called X-ray histotomography that can be used to assess the histology of whole zebrafish with cellular, and sometimes subcellular, resolution (Ding et al., 2019).

The zebrafish is a model organism that is widely used to study genotype-phenotype associations (Fuentes et al., 2018; Wangler et al., 2017) and human disease (Lieschke and Currie, 2007). Moreover, since zebrafish larvae and juveniles are small (less than 3 mm), they are ideal test subjects for potential 3D whole-organism imaging techniques (Figure 1A). One of the factors that can limit the performance of micro-CT is that a range of X-ray wavelengths are typically used to image the sample. Ding et al. overcome this problem by conducting their experiments at the Advanced Photon Source, a synchrotron that can produce highly focused X-rays over a narrow range of wavelengths, thus enabling monochromatic imaging (that is, imaging as a single wavelength), and imaging at beam energies that are orders of magnitude higher than commercial sources. The use of sophisticated software to control the exposure of the sample and to analyze the data they collect allows them to study whole zebrafish at both low and high resolution, with the potential to achieve high throughput rates for such studies.

**Figure 1.**
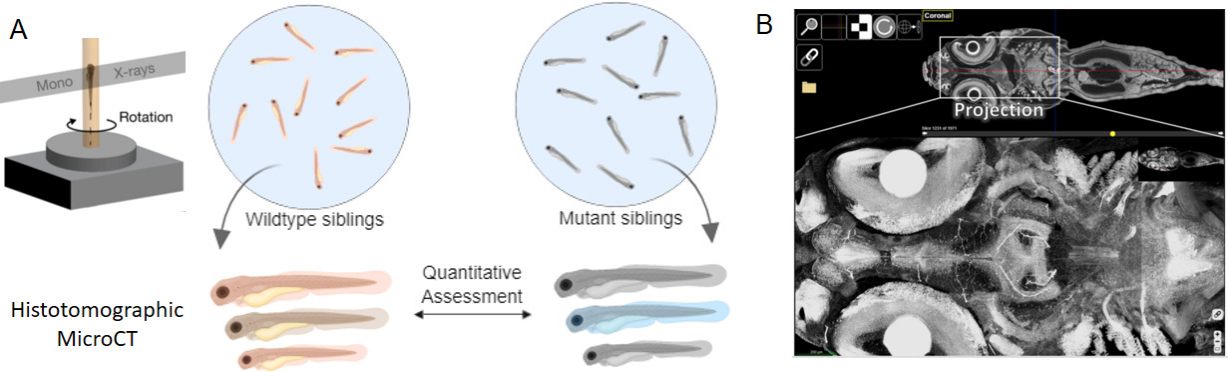
X-ray histotomography and phenotypic assessment in zebrafish. (**A**) At its most basic level, X-ray histotomography works by illuminating a fixed sample with a monochromatic beam of X-rays, and collecting the X-rays scattered by the sample as it is rotated. To achieve high resolution of entire organisms, Ding et al. use X-rays from a synchrotron radiation source (not shown); the scattered X-rays are converted into visible light by a scintillator and detected by a CCD camera (not shown). The combination of resolution and field of view offered by X-ray histotomography makes it possible to accurately characterize individual variations in both wild-type and mutant zebrafish at the subcellular level. (**B**) High-resolution image of a juvenile zebrafish (top), and an expanded view (bottom) showing details of the neural structure including individual axonal projections. This image is Figure 5—figure supplement 1 from Ding et al.

The researchers – who are based at Penn State, Duke University, the University of Chicago, Motorola Mobility and the Argonne National Laboratory – used X-ray histotomography to study both wild-type and mutant zebrafish. Ding et al. were able to identify a wide range of different cell types in a variety of organs – including neuronal cells in the eye and brain (Figure 1B), nucleated red blood cells in the heart, and goblet cells in the intestine – as well as various types of tissues. The technique can also readily identify significant morphological changes in zebrafish associated with both intrinsic factors (such as genetic mutations) and extrinsic factors (such as the presence of various molecules in the environment), thus showing that it has the potential to improve our understanding of the mechanisms by which genetic, environmental and other factors influence phenotype. Genetic background can also influence pharmacological and toxicological responses in zebrafish (reviewed in Sanders and Whitlock, 2003; Balik-Meisner et al., 2018; Brannen et al., 2010; Fuentes et al., 2018), so X-ray histotomography could also have an influence in these areas.

One of the biggest surprises was the high levels of variation observed in the wild-type specimens, even for sibling subjects. Previous analyses based on two-dimensional techniques had also suggested high levels of variation (Teixidó et al., 2019), but three dimensional techniques like X-ray histotomography have the potential to explore such phenomena in greater detail and in a more complete context. The vast amounts of data being produced by advanced imaging techniques are also redefining what is considered ‘normal’. The technique developed by Ding et al. has just begun to illuminate the many and varied ways in which a successful and functional organism can be formed. Understanding this range of normal – which may not be a normal distribution – is going to be essential to properly define 'abnormal'. As X-ray histotomography and other techniques get deployed at scale, we will surely uncover more surprises – in both mutant and wild-type contexts.
